# Interleukin-17A- or tumor necrosis factor α-mediated increase in proliferation of T cells cocultured with synovium-derived mesenchymal stem cells in rheumatoid arthritis

**DOI:** 10.1186/ar4355

**Published:** 2013-10-29

**Authors:** Zhengzheng Zhang, Yuanjing Ding, Weiping Li, Bin Song, Rui Yang

**Affiliations:** 1 Department of Orthopaedics, Sun Yat-sen Memorial Hospital, Sun Yat-sen University, 107 Yanjiangxi Road, Guangzhou 510120, PR China; 2 Department of Orthopaedics, Jinan Central Hospital, Shandong University, 105 Jiefang Road, Jinan 250013, PR China

## Abstract

**Introduction:**

Mesenchymal stem cells (MSCs) represent promising applications in rheumatoid arthritis (RA). However, the inflammatory niche in the RA synovium could adversely affect MSC function. This study was designed to investigate biologic and immunologic properties of synovium-derived MSCs (SMSCs) in RA, with particular focus on whether cytokines can mediate increase of proliferation of T cells cocultured with SMSCs in RA.

**Methods:**

Compared with SMSCs from eight healthy donors (HDs), SMSCs from 22 patients with RA (RAp) were evaluated. The methyl thiazolyl tetrazolium (MTT) assay was used to assess cell-population doubling and viability. Multipotentiality of SMSCs was examined by using appropriate culture conditions. Flow cytometry was used to investigate the marker phenotype of SMSCs. Immunomodulation potential of SMSCs was examined by mixed peripheral blood mononuclear cells (PBMCs) reactions, and then by PBMCs or synovial T cells with or without the addition of inflammatory cytokines (interleukin-17A (IL-17A), tumor necrosis factor-α (TNF-α), and interferon-γ (IFN-γ)) after stimulation with phytohemagglutinin (PHA), respectively.

**Results:**

SMSCs from RA patients (RA-SMSCs) showed normal population doubling, cell viability, multiple differentiation characteristics, and surface markers. In either mixed PBMC reactions or PBMC proliferation stimulated with PHA, RA-SMSCs showed normal immunomodulation function compared with SMSCs from healthy donors (HD-SMSCs). However, the increase in proliferation of T cells was observed when IL-17A and TNF-α were added alone or in combination.

**Conclusions:**

Our data suggest that the inflammatory niche, especially these cytokines, may increase the proliferation of T cells cocultured with SMSCs in RA.

## Introduction

Rheumatoid arthritis (RA) is a complex autoimmune disorder involved with multiple systems. Its characteristic is the destruction of cartilage and bone by the inflammatory mediators, such as interleukin-17A (IL-17A), tumor necrosis factor-α (TNF-α), and interferon-γ (IFN-γ). The etiology of RA is still under study, and multiple cells are thought to contribute to the pathogenic progression, in which T-cells [1] and fibroblast-like synoviocytes (FLSs) [2] are involved in a complex network leading to joint damage. Activation of Th1 cells and Th17 cells in the development of cell-mediated autoimmune arthritis has been investigated [3,4]. Conversely, Th2 cells and Treg cells maintain homeostasis in RA and in animal models of collagen-induced arthritis (CIA) [5,6].

Mesenchymal stem cells (MSCs) are multipotent progenitor cells. Although MSCs originally were isolated from bone marrow (BM), similar populations have been isolated from other tissues, including the synovial membrane [7], synovial fluid (SF) [8], tendon [9], periosteum [10], and joint fat [11]. These cells have the ability to differentiate into various other mesodermal cell lineages, including chondrocytes, adipocytes, and osteoblasts [12]. Another property of MSCs is their ability to inhibit the proliferation of multiple lymphocytes [13,14]. Because of their immunosuppression effects, MSCs represent promising applications in treatment of acute graft-versus-host disease [15]. However, the specific mechanisms by which bone marrow-derived MSCs (BMSCs) exhibit their immnoregulatory ability remain under discussion, and a difference is noted between the therapeutic effects for CIA models by MSCs [16,17]. The feasibility and safety of MSCs treatment have yet to be determined in larger cohort studies [18,19].

Recent studies have focused on an important role of synovium-derived mesenchymal stem cells (SMSCs) in local environment remediation [20,21]. It has been demonstrated that these processes contain direct recruitment of synovial cells into chondral defects [22] and their homing to injured sites [20]. With respect to RA, it is still important to consider the degree of the disease related to the inflammatory milieu, because inflammatory cytokines, such as such as IL-17A, TNF–α, and IFN-γ, have previously been shown to influence the functions of FLS and MSCs in the inflamed synovium [23]. Before contemplating clinical studies with MSCs in patients with RA (RAp), the proliferative and immunomodulatory capacity of SMSCs in this inflammatory condition must be explored. Inspired by the study of Farida Djouad and collegues [24], which revealed a reversal of immunosuppressive properties of MSCs by environmental parameters related to inflammation in CIA, we hypothesized that the immunomodulation function of SMSCs by IL-17A or TNF-α in RA should be reduced. Therefore, this study was designed to investigate biologic and immunologic properties of SMSCs in RA, especially focusing on whether cytokines can mediate the increase of proliferation of T cells cocultured with SMSCs in RA.

## Methods

### SMSCs from healthy donors (HD-SMSCs) and patients with RA (RA-SMSCs)

The study was approved by the Ethics Committee at Sun Yat-sen Memorial Hospital, and informed consent was obtained from all study subjects. Synovial tissue biopsies from the suprapatella pouch were obtained from 22 RAp and eight HD (For practical reasons, we chose patients with meniscus injury who were undergoing arthroscopy, and without any systemic immune disease or connective tissue disease, as the healthy donors) by using 3.5-mm grasping biopsy forceps under direct vision with arthroscopy. The RAp fulfilled the American College of Rheumatology criteria for RA [25]. The degree of macroscopic joint inflammation was evaluated by using the Visual Analogue Score (VAS). Scores (scaled between 0 and 100) were based on visual image of vasculature (redness and vessels due to hyperemia) in arthroscopy [26]. Synovial tissues were finely minced and digested with 0.4% collagenase (Gibco BRL Co.Ltd.,Gaithersburg, MD, USA) in high-glucose Dulbecco modified Eagle medium (DMEM) containing 10% fetal bovine serum (FBS), 100 U/ml penicillin, and 100 U/ml streptomycin. After overnight incubation at 37°C, cells were collected by centrifugation, washed twice, resuspended in high-glucose DMEM supplemented with 10% FBS, plated in a T25 culture flask, and allowed to attach for 7 days. Nonadherent cells were removed by changing the medium. Cells were passaged when reaching near confluence according to previous report [7].

### Peripheral blood mononuclear cells (PBMCs) isolation and synovial T-cells expansion

PBMCs were from HD and RAp by Ficoll-Hypaque density gradient (density, 1.077 g/L; Sigma). The clinical status of two groups is described in Table 1.

**Table 1:** Clinical status of RAp and HD

	**RAp**	**HD**
Number of Patients	22	8
Sex		
Men	11 (50%)	4 (50%)
Woman	11 (50%)	4 (50%)
Age (years)	42 (38 to 56)	40 (35 to 46)
Disease duration (years)	3.1 (0.2 to 5.5)	2.6 (0.1 to 4.6)
Treatment, patients (%)		
None	15 (68%)	7 (87.5%)
MTX	2 (9%)	-
MTX + SSZ	2 (9%)	-
NSAIDs	-	1 (12.5%)
Other^b^	3 (14%)	-
VAS	14 (5 to 35)	1 (0 to 2)

^b^Other treatments may have included corticosteroids, azathioprine, or leflunomide. In the HD group, disease duration refers to the duration of meniscus injury.

HD, healthy donor; MTX, methotrexate; Rap, patients with rheumatoid arthritis (RA); SSZ, sulfasalazine; NSAIDs, nonsteroidal antiinflammatory drugs; VAS, visual analogue score.

Synovial T cells were expanded from synovial tissues of RAp cultured for 14 days in RPMI-1640 medium supplemented with 10% FBS, in the presence of recombinant IL-2 (20 IU/ml; R&D Systems, Minneapolis, MN, USA). Cultures were fed every 3 days. T cells were then collected and analyzed with a cell sorter (FACS Vantage SE cell sorter; Becton Dickinson). More than 95% of the cells expressed CD3.

### SMSCs population doubling and viability test

SMSCs (P4) were vaccinated into 96-well plates at a concentration of 1 × 10^4^/ml, in a final volume of 100 μl fresh medium (10% FBS + high-glucose DMEM). For cell counting, three wells of each sample were digested by using 0.25% trypsin-ethylenediamine tetraacetic acid per day up to 12 days. With these data for cell-population doubling, we acquired the SMSC growth curves. With methyl thiazolyl tetrazolium (MTT, 5 mg/ml; Sigma), dimethylsulfoxide (DMSO; Sigma), and an EL800 microplate reader (BioTek Instruments, Winooski, VT, USA) that was to detect absorbance at 490 nm, we made the SMSCs viability curves in the same way, according to the day and the absorbance. Fresh medium was used as a negative control.

### In vitro differentiation potential assay

For the *in vitro* differentiation assays, three procedures (adipogenic differentiation, osteogenic differentiation, and chondrogenic differentiation) were used, as previous described [7]. The intracellular lipid accumulation as an indicator was visualized on day 21 with Oil Red O staining. The alkaline phosphatase (ALP) of SMSCs was assayed by using Cell ALP Staining assay (Nanjing Jiancheng, China), according to the recommendations of the manufacturers on day7 and alizarin red staining (AR-S, 1%, pH 7.2; Sigma) on day 28, respectively. The chondrogenic differentiations were confirmed with alcian blue staining. Meanwhile, the experimental controls were established by culture of SMSCs (P4) in fresh medium, and only fresh medium was used as a negative control. All measurements were tested in triplicate. An inverted phase-contrast microscope (Nikon Eclipse Ti-S; Nikon Corporation, Japan) visualized the images.

For quantitative assay, three procedures (adipogenic differentiation value, calcium deposits, alcian blue intensity) were used as previous represented [27,28].

### Immuno-phenotype of SMSCs

After treatment with 0.25% trypsin-ethylenediamine tetraacetic acid, SMSCs (P4) were then resuspended in PBS containing 0.5% BSA and 0.1% sodium azide. Cell aliquots (1 × 10^6^ cells/ml) were incubated on ice with conjugated mAbs against CD105, CD166, CD44, CD90, CD14, CD34, CD45, and HLA-DR (Table 2) or conjugated isotypic controls. Flow cytometry was performed on a FACScan laser flow-cytometry system (Becton Dickinson, San Jose, CA, USA), and data were analyzed with the CellQuest software (BD Bioscience, San Jose, CA, USA).

**Table 2:** Antibodies used to detect phenotype synovium-derived mesenchymal stem cells (SMSCs) with flow cytometry

**Antibody**	**Fluorochrome**	**Concentration (μg/ml)**	**Source**
CD14	PE	200	Santa Cruz Biotechnology, Inc. (Santa Cruz, CA, USA)
CD34	PE	50	Southern Biotech (Birmingham, AL, USA)
CD44	FITC	50	Becton Dickinson (Bedford, MA, USA)
CD45	PE	10	Caltag (Burlingame, CA, USA)
CD90	PE	1	Becton Dickinson (Bedford, MA, USA)
CD105	FITC	10	Becton Dickinson (Bedford, MA, USA)
CD166	FITC	50	AbD Serotec (MorphoSys, UK)
HLA-DR	PE	200	Santa Cruz Biotechnology, Inc. (Santa Cruz, CA, USA)

All antibodies were mouse IgG isotype antibodies obtained from commercial sources.

### Immunomodulation potential of SMSCs

The suppressive effects of SMSCs (P4) on mixed PBMCs reaction (MLR) and PBMCs proliferation stimulated by phytohemagglutinin (PHA) (4 μg/ml; Roche, Mannheim, Germany) were measured by using the MTT assay [29] and the ^3^H-TdR assay [30], as described previously. SMSCs were seeded in 96-well culture plates for 6 hours for adherence, and then irradiated (30 Gy) with Co^60^.

For the MLR, allogeneic PBMCs (15 × 10^4^ cells/cm^2^) from an HD were mixed with PBMCs from another unrelated HD in identical mounts. The mixed PBMCs were then mixed with different ratios (3 × 10^5^ cells/cm^2^ = 1:1 SMSC: PBMCs ratio, 15 × 10^4^ cells/cm^2^ =1:2, 6 × 10^4^ cells/cm^2^ =1:5, 3 × 10^4^ cells/cm^2^ = 1:10, 15 × 10^3^ cells/cm^2^ = 1:20) of SMSCs (experiment wells) or without SMSCs (blank wells) in 96-well culture plates to ensure efficient cell-cell contact for 5 days in 0.2 ml modified RPMI-1640 medium (Gibco) supplemented with 10% FBS.

The PBMCs proliferation assay only uses one autologous or allogeneic PBMCs reaction (30 × 10^4^ cells/cm^2^) from a healthy donor or patient with RA stimulated with PHA. Inhibitory or proliferative effects were measured on day 5 by using the MTT assay or the ^3^H-TdR assay. All measurements were performed in triplicate. Results were expressed as the mean (% inhibition or % proliferation) ± SD.

### Data analysis

Continuous variables were expressed as the mean ± SD, and categoric variables were presented as frequencies and percentages. The significance of the results was determined by using the unpaired Student *t* test, χ^2^ test, and repeated-measure tests with Bonferroni correction. Data analysis was performed with statistical software (SPSS, version 15.0 for Windows; SPSS Inc., Armonk, NY, USA). *P* < 0.05 was considered statistically significant.

## Results

### Population doubling and viability of SMSCs from RAp are normal

The RA-SMSCs growth curves (data not shown) have the same tendency as those for HD-SMSCs. The SMSCs population-doubling data of these two groups at each day (1 to 12 days) were tested by repeated-measures tests with Bonferroni correction, and the statistical result indicates that no statistically significant difference was present in SMSCs population doubling between RAp and HD (*P* = 0.157). For SMSCs viability at each point of time from 1 to 12 days, the difference of optical density (OD) at 490 nm between RAp and HD also was not statistically significant, as determined by cellular viability assays (*P* = 0.161).

### Triple differentiation potentials of RA-SMSCs *in vitro* were not changed

**Figure 1:**
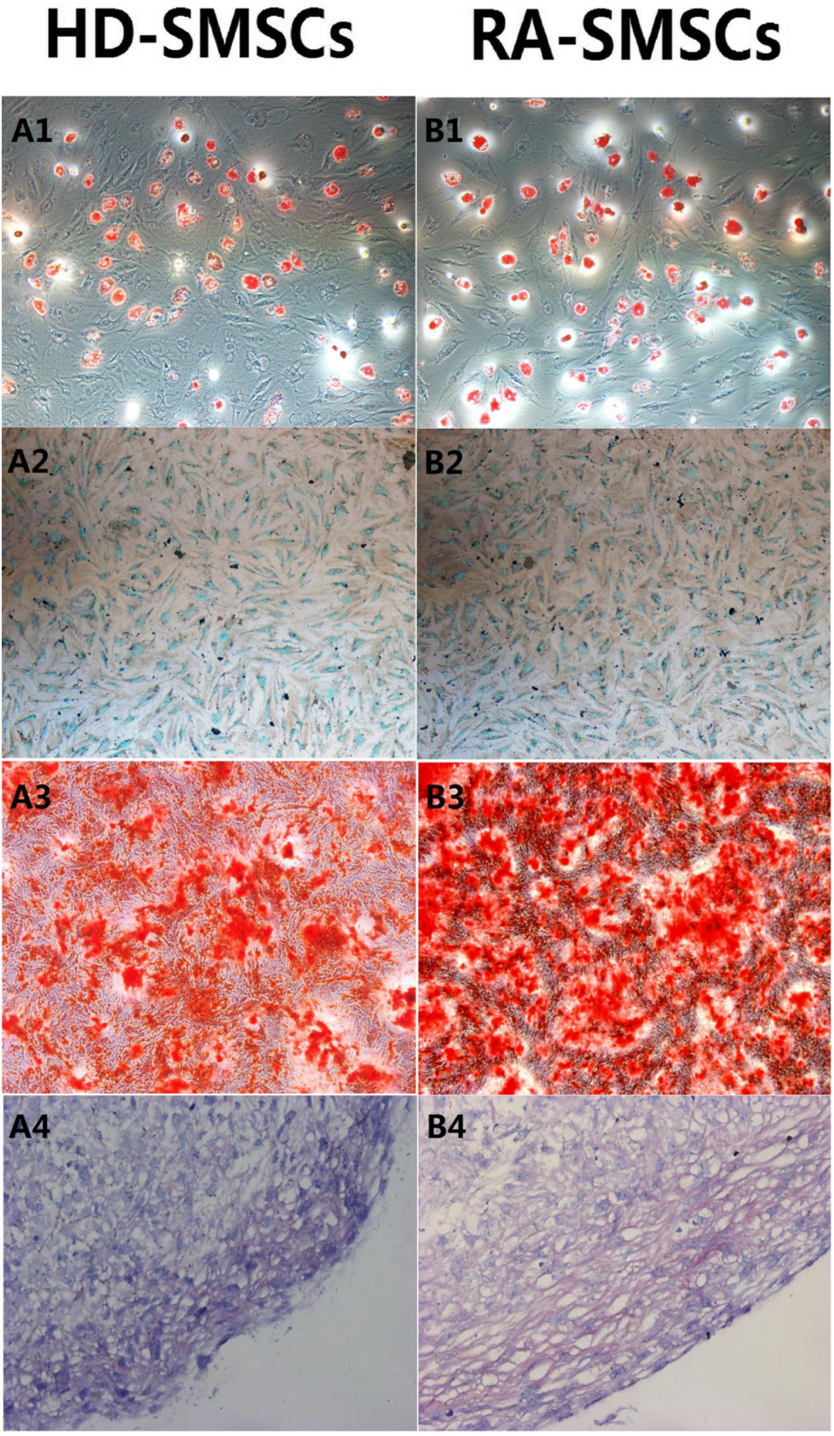
**Synovium-derived mesenchymal stem cells (SMSCs) triple differentiation potentials from healthy donors and patients with rheumatoid arthritis (RA). (A, B)** Morphologic characteristics of SMSCs for adipogenic, osteogenic, and chondrogenic differentiation evaluated with the inverted phase-contrast microscope; SMSCs from RA patients (RA-SMSCs) have the same morphologic properties as SMSCs from healthy donors (HD-SMSCs). Adipocytes were filled with many fat vacuoles, and Red Oil O was used to stain the fat vacuoles of adipocytes (**A1, B1**: ×100). Osteocytes were stained for alkaline phosphatase (ALP) with the Cell ALP assay (**A2, B2**: ×100) and for calcium deposition by using alizarin red-S (**A3, B3**: ×100). Chondroblast differentiation from SMSCs was displayed as cartilage-specific metachromasia with alcian blue (**A4, B4**: ×100).

In both groups, obvious differentiated adipocytes and osteocytes were detected as early as the day 14 after being induced for adipogenic and osteogenic differentiation, and obvious differentiated chondrocytes were seen at about 21 days since induction. For adipogenesis, the cells displayed the accumulation of lipid vacuoles, which stained with oil red O (Figure 1A1; B1). We analyzed the adipogenic differentiation value results by using IPP 6.0; no significant difference was found between RA-SMSCs and HD-SMSCs (*P* = 0.193). For osteogenesis, SMSCs from both populations treated with osteogenic medium underwent a change in their morphology from spindle shaped to cuboidal, and formed large nodules that stained for ALP and alizarin red (Figure 1A2, 3; B2, 3). For quantitative assay of mineral deposits, absorption of the dye at 562 nm revealed no significant difference in alizarin red-positive mineralized matrices (*P* = 0.088). For chondrogenesis, the cells displayed cartilage-specific metachromasia with alcian blue *in vitro* (Figure 1A4; B4). The alcian blue staining intensity of pellets in both groups had no significant difference (*P* = 0.075).

### Phenotypic characterization

**Figure 2:**
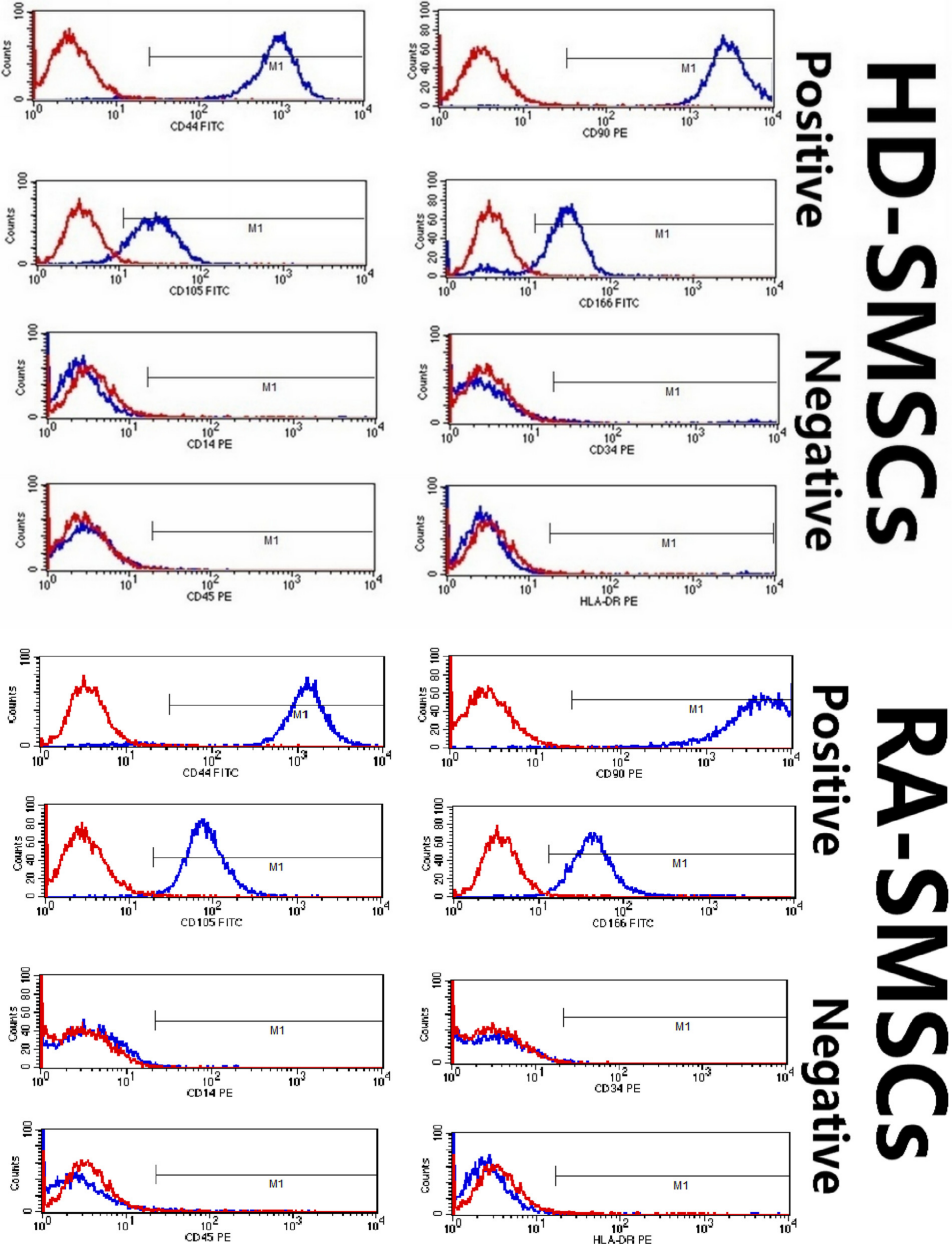
**Phenotyping of synovium-derived mesenchymal stem cells (SMSCs) for typical mesenchymal stromal cell-surface markers.** SMSCs from healthy donors (HD-SMSCs) and SMSCs from patients with rheumatoid arthritis (RA-SMSCs) were negative for the expression of CD14, CD34, CD45, and HLA-DR, and were positive for CD44, CD90, CD105, and CD166. Red lines indicate background fluorescence obtained with isotype control IgG. x axis, fluorescence intensity; y axis, cell counts.

By FACS analysis, for SMSCs from both tissues, the expression of CD14, CD34, CD45, and HLA-DR was negative, and the expression of CD44, CD90, CD105, and CD166 was positive (Figure 2). The expression intensity of each marker was not statistically different between the two populations (*P* > 0.05). Indeed, the cell populations isolated from two different tissues displayed a similar phenotype, just as the typical MSCs did [12].

### Normal suppressive potential of RA-SMSCs on either MLR or PBMCs proliferation stimulated with PHA

**Figure 3:**
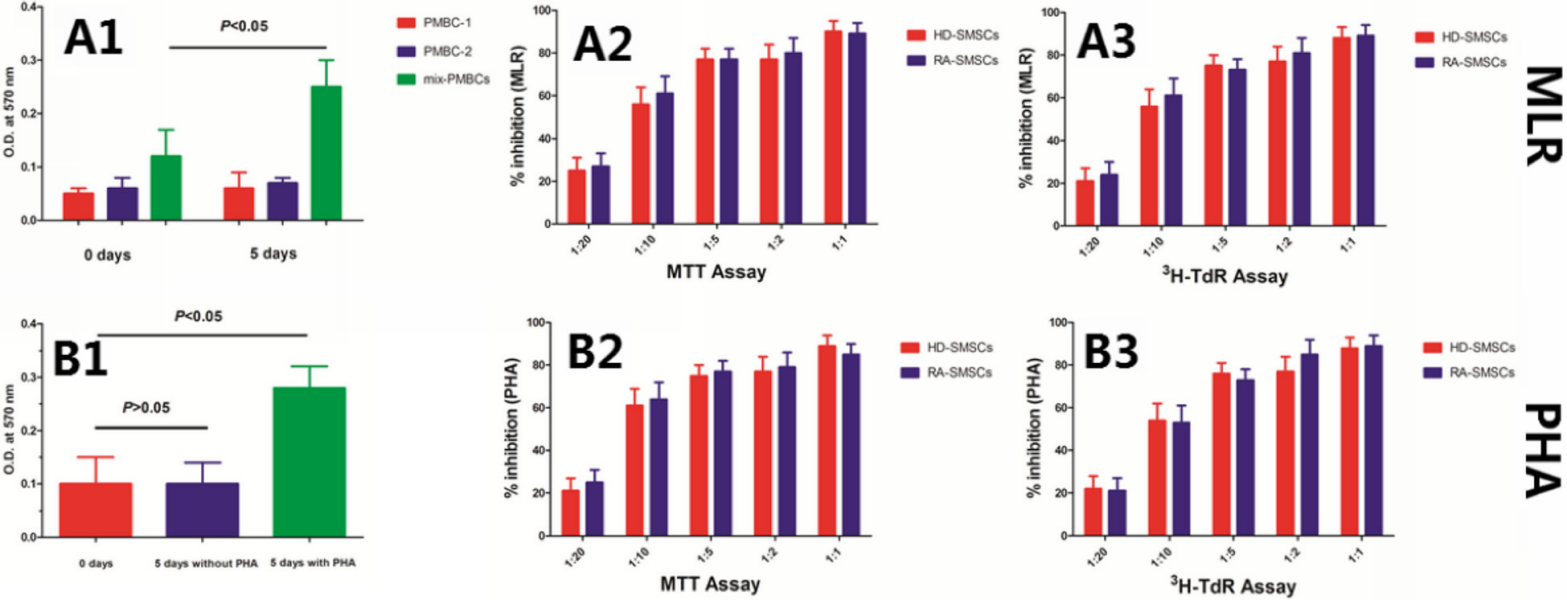
**Normal immunosuppressive potential of synovium-derived mesenchymal stem cells of patients with rheumatoid arthritis (RA-SMSCs). (A1)** The differences of absorbance between 0 days and 5 days were not statistically significant for the allogeneic peripheral blood mononuclear cells (PBMCs) from a healthy donor (*P* > 0.05) and the PBMCs from another unrelated healthy donor (*P* > 0.05); for the mixed PBMCs, however, the absorbance at 5 days was significantly higher than the value at 0 days (*P* < 0.05). **(A2)**, **(A3)** Compared with synovium-derived mesenchymal stem cells of healthy donors (HD-SMSCs), no statistically significant reduction in suppressive potential (% inhibition) on mixed PBMCs reaction (MLR) was found at all five ratios (*P* > 0.05). **(B1)** In autologous or allogeneic PBMCs proliferation assay, the differences of absorbance between 0 days and 5 days without PHA were not significant (*P* > 0.05), whereas the value for 5 days with PHA was significantly higher than the value at 0 days (*P* < 0.05). **(B2,B3)** Percentage inhibition of RA-SMSCs on PBMCs proliferation induced by PHA was similar with the values of HD-SMSCs at varied ratios (*P* < 0.05). Results were recorded as mean absorbance (optical density (OD)) ± standard deviation (SD) and as mean counts per minute (CPM) ± SD, respectively. The percentage inhibition values were calculated by using the following formulae: %inhibition = 1 − (OD(exp) − OD(adj))/OD(bla) or %inhibition = 1 − (CPM(exp) − CPM(adj))/CPM(bla). OD(exp), OD(adj) and OD(bla) represent the mean absorbance of experimental wells, adjusted wells (only SMSCs), and blank wells, respectively, and CPM(exp), CPM(adj), and CPM(bla) represent the mean counts per minute of the corresponding wells. Results were finally expressed as the mean (% inhibition) ± SD.

As shown in Figure 3, no statistically significant reduction in suppressive potential (percentage inhibition) of RA-SMSCs was found on MLR at all five ratios, compared with the percentage inhibition of HD-SMSCs (Figure 3A2, A3; *P* > 0.05). Similarly, RA-SMSCs produced no statistically significant decreased inhibitory effect on autologous or allogeneic PBMCs proliferation (Figure 3B2, B3; *P* > 0.05), ranging from a SMSC-to-PBMC ratio of 1:20 to 1:1. Furthermore, in either of them, the ^3^H-TdR assay data also suggested a significant relation between dose and inhibition of SMSCs from both populations was established.

### Involvement of IL-17A, TNF-α in the increase of the proliferation of T cells cocultured with SMSCs

To understand why SMSCs do not display any immunosuppressive effects in RAp, we tested the potential role of the inflammatory environment on SMSCs behavior. We therefore investigated the role of various cytokines (IL-17A, TNF-α, IFN-γ) on the interaction of synovial T cells from RAp with autologous SMSCs. Because the best ratio of SMSCs to PBMCs was 1:1, we used this cell ratio for subsequent experiments. Irradiated SMSCs and synovial T cells were cultured alone or were cocultured at a 1:1 ratio for 24 hours in the presence or absence of PHA (4 μg/ml), and exogenous IL-17A (50 ng/ml), TNF-α (50 ng/ml), or IFN-γ (50 ng/ml) was added either alone or in combination. Each experiment was performed in triplicate and repeated at least 3 times. The coculture of HD-SMSCs and synovial T cells from RAp were used as the experimental control.

**Figure 4:**
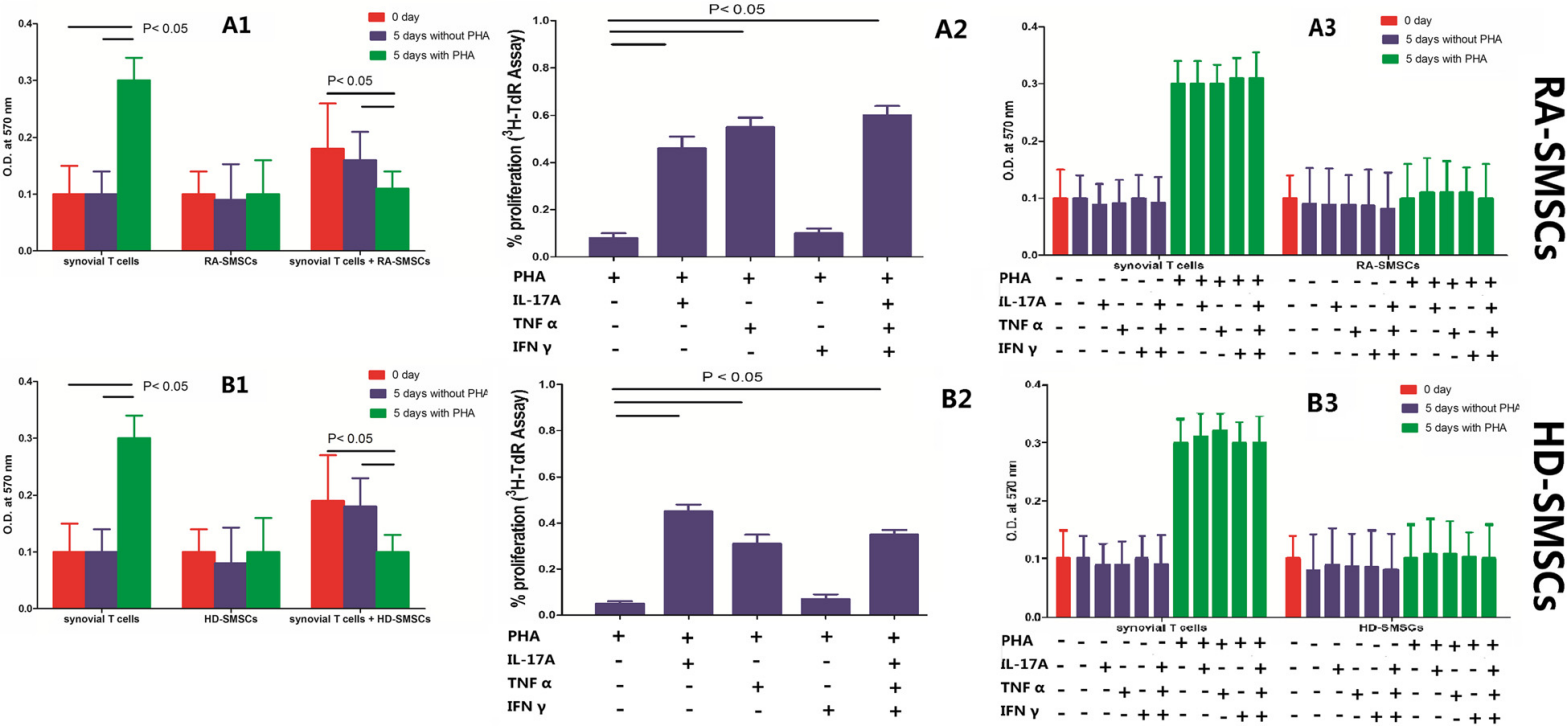
**Involvement of IL-17A, TNF-α in the increase of the proliferation of T cells co-cultured with SMSCs. (A1, B1)** In synovial T cells proliferation assay, the differences of absorbance between 0 days and 5 days without PHA were not significant (*P* > 0.05), whereas the value for 5 days with PHA was significantly higher than the value at 0 days (*P* < 0.05); in SMSCs proliferation assay, no differences were noted between 0 days and 5 days with or without PHA; in the coculture of synovial T cells and SMSCs, the absorbance for 5 days with PHA was significantly lower than the value at 0 days or 5 days without PHA (*P* < 0.05). **(A2, B2)** In synovial T cells activated by PHA and SMSCs cocultured in the presence of cytokines assay, the increase of the proliferation (% proliferation) of T cells was significant in the presence of IL-17A or TNF-α (*P* < 0.05). However, no effect was observed when IFN-γ was added (*P* > 0.05). In addition, the increase of proliferation was significant when cytokines were added in combination (*P* < 0.05). **(A3, B3)** IL-17A and TNF-α had no effect on RA-SMSC or synovial T-cells cultured alone at 5 days with or without PHA (*P* > 0.05). Results were recorded as mean absorbance (optical density (OD)) ± standard deviation (SD) and as mean counts per minute (CPM) ± SD, respectively. The percentage proliferation values were calculated by using the following formulae: % proliferation = (OD(exp) − OD(adj))/OD(bla) or % proliferation = (CPM(exp) − CPM(adj))/CPM(bla). OD(exp), OD(adj), and OD(bla) represent the mean absorbance of experimental wells, adjusted wells (only SMSCs), and blank wells, respectively, and CPM(exp), CPM(adj), and CPM(bla) represent the mean counts per minute of the corresponding wells. Results were finally expressed as the mean (% proliferation) ± SD.

As shown in Figure 4A1, RA-SMSCs totally inhibited the autologous response of synovial T-cells (*P* < 0.05). In contrast, the proliferation of synovial T cells was increased when cultured with RA-SMSCs in the presence of IL-17A or TNF-α (*P* < 0.05; Figure 4A2). IL-17A and TNF-α had no effect on RA-SMSC or synovial T cells cultured alone (*P* > 0.05; Figure 4A3). No effect was observed when IFN-γ was added to the reactions (*P* > 0.05). Moreover, HD-SMSCs were unable to suppress the proliferative response in the presence of TNF-α and IL-17A, either alone or in combination (Figure 4B; *P* < 0.05).

## Discussion

We demonstrated that RA-SMSCs showed normal population doubling, cell viability, multiple differentiation characteristics, and surface markers; and also in either MLR or PBMCs proliferation stimulated with PHA, RA-SMSCs showed normal immunomodulation function compared with HD-SMSCs. Impressively, the increase of proliferation of T cells cocultured with SMSCs was observed when IL-17A and TNF-α were added alone or in combination.

Although recent studies suggested that the imbalance of Th17/Treg cells plays a crucial role in the progression of RA [31], the mechanisms leading to their study in the RA synovium remain unknown. Moreover, even through some cytokines, especially IL-6, IL-23, and transforming growth factor β (TGF-β), which facilitate the differentiation of Th17 [32], are demonstrated in the RA synovium; the other cytokines, such as IFN-γ, which counteract their differentiation, are also found [1,2]. In addition, the immune regulation of T cells by MSCs has also been demonstrated [7,33]. Although previous studies have assumed that the functional deficiency of reg cells in RA may be the reason [34], others have proposed that the interaction of BMSCs with T cells promotes the activation and expansion of Th17 cells [35]. In conclusion, among the cytokines produced by FLSs and synovial T cells, IL-17A, TNF–α, and IFN-γ have been found to play pivotal roles in RA [3,4,36]. We thus investigated whether these cytokines could influence the immunosuppressive properties of SMSCs.

To date, only a few studies have explored MSCs in RA, and these were focused on BMSCs [35,37]. To our knowledge, our study is the first investigation describing the interaction between the inflammatory niche and RA-SMSCs. In RA, *in viv*o MSCs showed an inflammation-related reduction in numbers. Extensive proliferation leading to synovial hyperplasia could explain this reduction [38]. Clonal BMSCs from RAp (RA-BMSCs) were more heterogeneous in their proliferative capacity and, on average, grew more slowly than clonal BMSCs from patients with osteoarthritis. This could be explained by variable premature telomere shortening previously observed for RA-BMSCs [37]. Our data suggest that RA-SMSCs showed normal biologic characteristic, such as cell-population doubling, cell viability, multiple differentiation, and surface markers, compared with HD-SMSCs. Previous study has suggested a negative relation between SMSC chondrogenic and clonogenic capacities and VAS in RA [39], and our study found a negative relation between alcian blue intensity and VAS (data not show) could explain normal biological property. As VAS of RAp in our study was low (Table 1), chondrogenic capacities may be affected by this degree of clinical status. Moreover, previous SMSCs were isolated from synovial tissues at the time of arthroplasty for degenerative OA or RA [40], and the VAS of these patients was almost as high.

Although RA-BMSCs seem to be similar to normal BMSCs, in that they can also inhibit the proliferation of autologous and allogeneic PBMCs *in vitro*[41], Evangelia Yannaki [42] demonstrated that BMSCs lose their immunomodulatory properties when infused in the inflammatory micromilieu of RA. They found conditioning of the recipient with bortezomib alters the disease microenvironment, enabling BMSCs to modulate arthritis. The discrepancy of MSCs function between *in vitro* and *in vivo* conditions, implies that a common endogenous stimulus may alter the *in vivo* performance of MSCs and that the immune privilege ascribed to MSCs may be susceptible to the influence of the microenvironment. We showed that the increase of proliferation of T cells cocultured with SMSCs was observed when IL-17A and TNF-α were added alone or in combination *in vitro*. Thus, we speculated that those cytokines may influence the immunosuppressive properties of SMSCs in RA. *In vitro*, failure of SMSCs to induce immunomodulation in severely inflammatory conditions may reflect the dynamic nature and complex network of specific microenvironments *in vivo*.

The major limitation of the present study is that we did not investigate the mechanism by which IL-17A and TNF-α affect proliferation of T cells. Although previous study agree with an effect on immunosuppressive properties of MSCs [24], but a direct effect on T cells, rendering them resistant to suppression, cannot be excluded. Further research is required.

## Conclusion

Our findings demonstrate the existence of SMSCs in RA and the inflammatory micromilieu of RA increases the proliferation of T cells cocultured with SMSCs. This information furthers our understanding of the etiology of RA, and strategies to alter the inflammatory milieu before SMSC action may be of critical importance.

## Abbreviations

ALP: Alkaline phosphatase; BM: Bone marrow; BMSC: Bone marrow-derived mesenchymal stem cell; CIA: Collagen-induced arthritis; DMSO: Dimethyl sulfoxide; FBS: Fetal bovine serum; FLS: Fibroblast-like synoviocyte; HD: Healthy donor; HD-SMSCs: Synovium-derived mesenchymal stem cells from healthy donors; IFN-γ: Interferon-γ; IL-17A: Interleukin-17A; MLR: Mixed peripheral blood mononuclear cells reaction; MSC: Mesenchymal stem cell; MTT: Methyl thiazolyl tetrazolium; PBMC: Peripheral blood mononuclear cell; PHA: Phytohemagglutinin; RA: Rheumatoid arthritis; RAp: Patient with RA; RA-BMSCs: Bone marrow-derived mesenchymal stem cells from RA patients; RA-SMSCs: Synovium-derived mesenchymal stem cells from RA patients; SF: Synovial fluid; SMSCs: Synovium-derived mesenchymal stem cells; TGF-β3: Transforming growth factor-β3; TNF-α: Tumor necrosis factor-α; VAS: Visual analogue score.

## Competing interests

The authors declare that they have no competing interests.

## Authors’ contributions

ZZ and YD performed the study, analyzed and interpreted the data, and drafted the article. BS participated in the design and coordination of experimental work; RY and WL designed the study and revised the article. All authors read and approved the final manuscript.

## Authors’ information

Zhengzheng Zhang and Yuanjing Ding are the co-first authors.
